# 2-octyl cyanoacrylate versus reintervention for closure of urethrocutaneous fistulae after urethroplasty for hypospadias: a randomized controlled trial

**DOI:** 10.1186/1471-2490-14-93

**Published:** 2014-11-21

**Authors:** Gabriela Ambriz-González, Pedro Aguirre-Ramirez, José Manuel García-de León, Francisco Javier León-Frutos, Sergio Adrián Montero-Cruz, Xóchitl Angélica Rocío Trujillo-Trujillo, Clotilde Fuentes-Orozco, Michel Dassaejv Macías-Amezcua, Andrea del Socorro Álvarez-Villaseñor, Ana Olivia Cortés-Flores, Mariana Chávez-Tostado, Alejandro González-Ojeda

**Affiliations:** Pediatrics Surgery Department, Medical Unit of High Specialty, Pediatrics Hospital of the Western Medical Center, Mexican Institute of Social Security, Guadalajara, Jalisco México; Pediatrics Urology Department, Medical Unit of High Specialty, Pediatrics Hospital of the Western Medical Center, Mexican Institute of Social Security, Guadalajara, Jalisco México; Private practice, Guadalajara, Jalisco México; Universitary Center of Biomedical Research, University of Colima, Colima, Colima, México; Research Unit in Clinical Epidemiology, Medical Unit of High Specialty, Specialties Hospital of the Western Medical Center, Mexican Institute of Social Security, Avenida Belisario Domínguez 1000, Colonia Independencia, CP 44340 Guadalajara, Jalisco México; Coordination of Health Research, Mexican Institute of Social Security, La Paz, Baja California Sur México

**Keywords:** Hypospadias, Urethrocutaneous fistula, 2-Octyl cyanoacrylate, Fistula repair

## Abstract

**Background:**

Urethrocutaneous fistulae (UCFs) represent one of the most frequent causes of morbidity after urethroplasty. Hypospadias can be repaired using different surgical techniques, but—regardless of technique—the incidence of UCF ranges between 10% and 40%. Surgical repair of UCF remains the treatment of choice, even if some patients need further surgery because of recurrences. Cyanoacrylates have been used as skin suture substitutes, and some evidence suggests a beneficial effect when these adhesives are used as an adjuvant in the management of UCF. Here we describe the results of management of UCF using 2-octyl cyanoacrylate (OCA) compared with surgical repair.

**Methods:**

A randomized clinical trial conducted from January 2008 to December 2012 included 42 children with UCF complications after urethroplasty for hypospadias. Twenty-one children were assigned to receive OCA as ambulatory patients and 21 were treated surgically. The main outcome variable was closure of the UCF. The estimated costs of both treatments were also calculated, as were absolute risk reduction (ARR), relative risk reduction (RRR) and number needed to treat (NNT) to prevent a surgical intervention.

**Results:**

The mean numbers of UCF were 1.3 in the OCA group (n = 28) and 1.1 in the surgical group (n = 25) with no statistically significant difference. The external orifices measured were 2.96 ± 1.0 mm and 3.8 ± 0.89 mm, respectively (NS). Sixty per cent of the UCFs treated with cyanoacrylate were completely closed and 68% of the surgical group healed completely (NS). More than one reoperation to improve complications was needed in the surgical group (3.5 ± 1.2). The clinical significance of the therapeutic usefulness of OCA was demonstrated by an ARR of 0.08, RRR of 0.25 and NNT of 12 to avoid further surgical treatment. The total costs of adhesive applications and reoperations were $US 14,809.00 and $US 158,538.50, respectively.

**Conclusions:**

The results showed a similar success rate for both treatments. However, sealant use should be considered before surgical treatment because this is a simple outpatient procedure with a reasonable success rate.

**Trial registration:**

ClinicalTrials.gov Identifier: NCT02115191. Date: April 13, 2014.

## Background

Hypospadias is a urological birth defect in which the urethral meatus is located ventrally anywhere from the tip of the glans up to the perineum [1]. These defects occur in 1 in 300 live births (0.3%). Kallen et al. reported a prevalence of 0.26 cases per 1000 births in Mexico, 2.6 per 1000 births in Hungary and about 5.2 per 1000 births in Scandinavia, and annually about 6000 neonatal boys in the US are diagnosed with hypospadias [2]. The anomaly is detected during examination at birth or prior to the completion of circumcision [3]. Nine per cent of cases are associated with inguinal hernia [1, 3]. Hypospadias repair was first described in 100–200 years BC; since then, more than 200 repair techniques have been described and new techniques are being developed continuously [3]. Initially, hypospadias surgical repair (HSR) was done in stages, but since 1950, a one-stage repair has been considered the treatment of choice [4]. The incidence of complications varies depending on the severity of the hypospadias, the patient’s age, previous attempts to repair and tissue condition. Complications can be immediate, including wound infection and haematoma. Late complications include meatal stenosis and retraction, particularly in the urethra, dehiscence, stricture and diverticulum, and a urethrocutaneous fistula (UCF). A UCF is very common and requires reoperation with a 6–12-month interval between each procedure and a high risk of failure when simple procedures are used [5–10]. Many cyanoacrylate-based adhesives have been studied, including isobutyl cyanoacrylate, isohexyl cyanoacrylate and 2-octyl cyanoacrylate (OCA). These adhesives have tensile strength, and bacteriostatic and haemostatic properties. N-Butyl-2-cyanoacrylate (NBCA) and OCA are the most frequently used, but the latter has been shown to have better tensile strength, equivalent to a suture after 7 days [10–13]. NBCA has been used in the urological field for the embolization of arteriovenous fistulae, in renal segmental embolization, to seal the vas deferens in sterilization, as a method of resolving urinary fistulae, and instead of sutures during circumcision [12–17]. In 2002, Lapointe et al. reported a case series of UCF management with NBCA, which evaluated the efficacy and safety in eight patients, achieving fistula closure (FC) with adequate functionality and aesthetics in five (63%). The others had a remaining fistula and were treated with a second application of NBCA combined with bladder drainage, and subsequently underwent surgical repair that was successful in all of the patients [18]. However, no studies have been designed to test the utility of using cyanoacrylate derivatives, especially OCA, as an alternative to traditional surgical treatment of UCF in children. Here we compared the surgical treatment for closure of UCF secondary to HSR that is used at our institute with the application of OCA, and analysed whether this adhesive could be an effective alternative treatment that promotes the closure of UCF. The aim was to evaluate the effectiveness of OCA to treat UCF and avoid reoperations.

## Methods

From 1 January 2007 to 31 December 2012, a double-blind randomized clinical trial was conducted in children with cases of UCF that occurred after hypospadias repair. The patients were assigned to either the control or the study group. The study group included children with long-standing UCF that appeared at least 6 months after the initial intervention. After preparation of the fistula area, multiple layers of OCA were applied, attaching the edges of the orifice. The same procedure was repeated three times and children were followed for at least 12 months after treatment. The control group included children with long-standing UCF that occurred at least 6 months after the initial intervention. Patients were scheduled for surgical treatment and followed for at least 6 months after surgery. If UCF recurred, a new intervention was proposed. The sample size was determined based on a prevalence of the complication (UCF) of 25% in the surgery group and 75% in those patients treated without any adjuvant or adhesive treatment and considered as having attained spontaneous closure (25%), given a confidence level of 95% and a power of 0.20, which resulted in a minimum sample size of 18 patients per group. The inclusion criteria included children younger than 5 years old with at least one postoperative UCF with an external orifice less than 5 mm in diameter at the distal, proximal or medial penis, and whose parents or guardians accepted inclusion in the study by signing the informed consent form. Patients were excluded for the following reasons: if the external orifice was more than 5 mm in diameter; if there were iatrogenic or traumatic fistulae; the presence of obstruction distal to the fistula; if there was necrotic tissue or active infection at the surgical site; if they had chronic diseases such as diabetes mellitus, renal or hepatic insufficiency, malignancy of any type; or the use of steroid and/or immunosuppressive therapy of any kind. The independent variables were the application of OCA to the UCF orifice or surgical treatment of UCF, and the main outcome variable was FC without recurrence within at least 12 months of follow-up. As confounding variables, we considered the children’s age, the fistulous orifice diameter, and the type of initial surgical procedure to which all were submitted.

### Procedure

Assignment of patients to groups was performed using a sealed envelope chosen randomly by the father, mother or guardians. These were prepared by a third person not involved in the protocol and kept in an agreed location. Follow-up staff and data analysts were kept blinded to each patient’s allocation. To decrease the inflammatory response and oedema, patients in the study group were instructed to apply triamcinolone cream to the fistula four times a day for 3 days. Once this treatment was completed, the patient attended our clinic as an outpatient to undergo treatment, after sedation with midazolam (0.5 mg/kg orally) and Foley catheterization with a 10 Fr gauge siliconized catheter, with the balloon inflated with 1.5 ml of water to prevent bladder spasms. After preparation of the fistula area with a gentle scarification of the edges with a 27-g needle and cotton swabs to produce slight bleeding, we applied OCA adhesive using Adson dissecting forceps to approximate the edges of the UCF and then applied several thin layers of OCA. The urethral catheter was left in place for 5 days. At the end of this time, we evaluated any closure or persistence of the UCF. If the UCF persisted, the OCA adhesive was applied twice more at intervals of 30 days following the same protocol, before the medical procedure was deemed to have failed. Patients in the control group waited 6 months to be treated surgically. The reoperation consisted of mobilizing dartos flaps and closing the defect in layers with absorbable sutures. Urinary bladder drainage was provided with a 10 Fr silicone Foley catheter, with the balloon inflated with 1.5 ml of water to prevent bladder spasm. The follow-up time was 12 months for each group.

### Statistical analysis

The descriptive phase of the analysis included the presentation of data as raw proportions, and means and standard deviations. Fisher’s exact test or the χ² test were used to compare qualitative variables. The distribution of the quan- of the quantitative variables was analysed with the Kolmogorov Smirnov test to determine whether they showed a normal distribution as required for the use of Student’s *t* test. Microsoft Excel 2007 (Redmond, WA, USA) and SPSS for Windows (version 17; SPSS Inc., Chicago, USA) were used for data processing and statistical analysis. For all variables, a value of P <0.05 was considered statistically significant. To establish the clinical significance of the results, we determined the absolute risk reduction (ARR), relative risk reduction (RRR) and number needed to treat (NNT) to avoid reoperation.

### Ethical considerations

This study was conducted according to the Declaration of Helsinki (2008) and Mexican Health Guidelines. The protocol was approved by the Ethics Committee of the Children’s Hospital of the Western National Medical Center (code 2003-1202-036). Also, the protocol was registered at National Clinical Trials number 02115191. Full written informed consent was obtained from all patients’ carers before their inclusion in the study.

## Results

**Figure 1:**
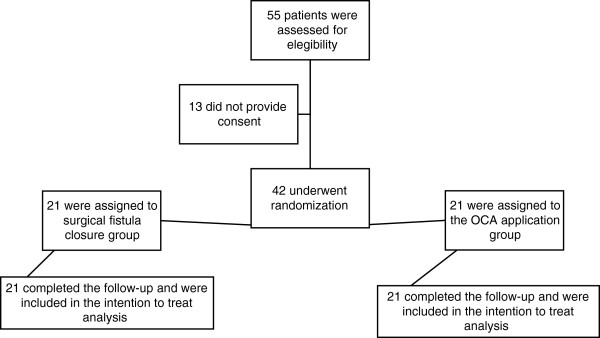
**Enrolment and outcomes.**

From 1 January 2007 to 31 December 2012, 42 children who had one or more UCFs following surgical treatment for hypospadias were enrolled (Figure 1). Twenty-one children were assigned to group 1 (application of OCA) and 21 were assigned to group 2 (surgical closure). The average age of group 1 was 28.6 ± 24.5 months (range 10–76 months) and that of group 2 was 35.4 ± 17.6 months (range 9–82 months; NS). Based on the Barcat classification of hypospadias, group 1 included nine patients (43%) with proximal, seven (33%) with medial and five (24%) with distal hypospadias; group 2 included 10 patients (48%) with proximal, six (29%) with medial and five (24%) with distal hypospadias. There was no significant difference between the groups in the location of the hypospadias. Congenital malformations were present in nine patients (43%) in group 1 and in 13 patients (62%) in group 2; this was not significantly different as described in Table 1. In group 1, the average number of UCFs per patient was 1.3 (range 1–4), while for group 2 it was 1.1 (range 1–2; NS). No significant difference was found between groups in the size of the UCFs; the average size in group 1 was 2.96 ± 1.0 mm compared with 3.82 ± 0.89 mm in group 2 (NS). The characteristics of both groups related to the surgical technique used for the initial cure of hypospadias prior to inclusion in this study are presented in Table 2. In group 1, 17/28 successful UCF closures were achieved without recurrence with an average of 2.5 applications of 1 ml of OCA (range 1–3), while in group 2, 17/25 UCF closures were achieved with an average of 3.2 ± 1.2 surgeries. There was no significant difference between the groups in the rate of resolution of the UCFs (Table 3). However, the clinical significance of the therapeutic usefulness of OCA was demonstrated by an ARR of 0.08 (8%), RRR of 0.25 (25%) and an NNT of 12 to avoid surgical treatment. Although it was not an objective of this study to perform a cost–benefit analysis, we found that for medical treatment, the cost of each adhesive application was 3,526 Mexican pesos (equivalent to $US 282), while the cost of surgery and a 1-day hospital stay was 28,668 Mexican pesos ($US 2,294). The total cost of treating each group, considering the total number of applications of OCA and reoperations to achieve a successful closure of the UCF, was 185,115 Mexican pesos ($US 14,809) for group 1 and 1,931,731.20 Mexican pesos ($US 158,538.50) for group 2. Reported complications resulting from the application of the OCA adhesive were minor: nine patients reported a sensation of local temperature increase (42.8%), and 2 days of transient dysuria was reported in one patient (4.7%).

**Table 1:** **General characteristics of patients included in the protocol**

Characteristic	Study group n = 21	Control group n = 21	P
Age (months)	28.6 ± 24.5	35.4 ± 17.6	NS*
Barcat classification			NS**
Proximal	9 (43%)	10 (48%)	
Medial	7 (33%)	6 (29%)	
Distal	5 (24%)	5 (24%)	
Number of fistulae	1.3 (1–4)	1.1 (1–2)	NS*
Diameter (mm)	2.96 ± 1.0	3.82 ± 0.89	NS*
Congenital malformations	9 (43%)	13 (62%)	NS_**_
Inguinal or umbilical hernia	4	5	
Cryptorchidism	3	6	
Cleft lip and palate	1	1	
Persistence of arterious conduct	1	1	

NS, not significant; *Student’s *t* test; **χ2 test.

**Table 2:** **Surgical technique employed to treat hypospadias**

Surgical technique	Study group n = 21	Control group n = 21
Duckett	3	3
MAGPI	5	3
Onlay	3	1
Snodgrass	9	9
Thiersch–Duplay	1	5
**Total**	**21**	**21**

MAGPI, meatal advancement and glanuloplasty technique.

**Table 3:** **Main results in both groups of patients**

	Study group n = 21	Control group n = 21	P
Number of UCFs	28	25	NS
Interventions to close UCF	2.5 ± 0.5*	3.2 ± 1.2**	NS
Successful UCF closure	17/28 (60%)	17/25 (68%)	NS, 0.86 (0.52–1.44)***

NS, not significant. *Number of applications of 2-octyl cyanoacrylate. **Number of surgical interventions. ***Relative risk (95% confidence interval).

## Discussion

The management of UCF resulting from surgery to cure hypospadias remains a problem, as the rate of this complication is high: the average reported incidence is 35% [6–9]. In a cohort study conducted at our hospital, the incidence of UCF was 41% in children undergoing surgical repair of hypospadias. The incidence was notably different from that (10%) observed in patients undergoing similar surgical procedures that included the application of fibrin glue with or without reinforcing suture lines at the surgical site [19]. In contrast, Barbagli et al. evaluated 1176 patients undergoing treatment for hypospadias with different techniques. They observed complications in 64.6% of their patients, of which UCF was the most common (20.7%). They used different approaches to perform the closure, and the number of reoperations was 2–23 per patient (mean 5) [6]. The surgical technique used to repair hypospadias has a definite impact on the development of UCF. Thus, the incidence of UCFs with an advanced technique of urethroplasty was 0.5–10%, in the graft wrap technique it was 2.2–35%, for the distal urethral hypospadias advancement it was 1–16.7% and in tubulization with pedunculated island repair it was 4–33% [20–24]. Smith reported that the incidence decreased to less than 3% with the two-stage technique of repair [25], and Greenfield et al. reduced the incidence to less than 2.5% using the modified Belt–Fuqua two-stage technique in cases of severe hypospadias [26]. However, reports persist of adverse outcomes after initial surgery for hypospadias corrections, with a prevalence of 55–77% for UCF [27, 28]. Cyanoacrylates are monomers that polymerize quickly when hydrogen ions are present, creating a solidified acrylic resin in less than a minute. They exist in two forms: as short-chain cyanoacrylates including methyl-2-cyanoacrylate or ethyl-2-cyanoacrylate, which are rarely used because of their rapid degradation to formaldehyde, a powerful tissue toxin; and as long-chain cyanoacrylates including NBCA or OCA, which degrade more slowly and have lower toxicity, and so are widely used in the repair of facial skin wounds [29–31]. Bardari et al. [32] noted that the use of the cyanoacrylate family of adhesives is an excellent option for the management of urinary fistulae before deciding on surgical treatment, because of its safety and effectiveness with only minor risks. In a small study, Lapointe et al. [18] established the utility of using NBCA to heal UCF, with success in five of eight patients (63%), and alluded to a savings in resources with the use of the cyanoacrylate in contrast to the higher cost of surgery. In 2011, Prestipino et al. [33] reported a small clinical trial using NBCA, including six children with early development of UCF (<72 h after urinary catheter removal following HSR) and seven children with late UCF development (up to 6 months after HSR), noting successful FC in seven of the 13 children (54%). Based on this, even though they concluded that surgical repair of UCF is the gold standard, they emphasized the ease of adhesive application even without sedation, which could be repeated several times as needed: the principal advantage was increased tensile strength. They also proposed a clinical trial to confirm whether surgical treatment should remain the gold standard. Tan et al. [34] reported a retrospective case series, including 37 children with UCF after HSR in whom at least one attempt at repair had been made, who underwent surgical treatment according to the location of the fistula. Tubulized urethroplasty was performed in eight patients; 17 of those with proximal hypospadias and cord presence needed a phased repair by initial cord fixation followed by urethroplasty; and 12 patients with multiple UCFs who, despite previous repair attempts, underwent urethroplasty, urethral diverticulum resection and tubulization as required on an individual basis. All patients had suture line reinforcement with OCA. The study found UCF recurrence in only four (11%) of the patients. Based on the waterproofing and moisture-resistance properties of the cyanoacrylate adhesives, Hosseini et al. [35] undertook a case–control study in 61 children with a mean age of 13.5 months who underwent HSR using different techniques. Patients were divided into two groups: 41 children in whom conventional dressings were placed on the surgical site (control group) against 20 children in whom the suture lines were reinforced with NBCA+OCA (study group). The authors observed that the prevalence of bruising and infection was 10% in the study group and 25% in the control group. They emphasized the beneficial effects of decreasing the incidence of haematomas and infections from exposure to urine and faeces in children treated with the waterproof adhesive. Our study here was robust statistically because of the number of patients included and the randomization procedure for the application or non-application of OCA. We demonstrated that UCF closure was achieved in 60% of our adhesive-treated UCF patients and in 68% of those treated surgically. Although the difference between groups in the successful UCF closure rate was not statistically significant, these results indicate clearly that the procedure should be included in the postoperative management of UCF and, as suggested by Lapointe et al. [18], Prestipino et al. [33], Tan et al. [34] and Hosseini et al. [35], should be regarded as an alternative to surgery for preventing or treating UCF. These observations support the results of simpler studies, such as those reported by Tsur et al. [36] and Castañón García-Aliz et al. [37]. It is important to note that the cyanoacrylate closure manoeuvre can be attempted on numerous occasions and, as suggested by Prestipino et al. [33], even in the absence of sedation. In contrast, our results with the gold standard surgical treatment showed that success was achieved only after an average of three reoperations. The studies reported in the literature suggest that the occurrence of major adverse side effects that require removal of different types of adhesive (NBCA, OCA or NBCA plus OCA) are rare. Our evaluation showed that the most common side effect with OCA was a feeling of increased temperature without burning or necrosis.

## Conclusions

These adhesive products may be considered an excellent alternative medical treatment that can be performed on an outpatient basis without sedation or anaesthesia. It causes less psychological stress or anxiety than the surgical management of children, which requires hospitalization and has some inherent risks. Other advantages are that the use of adhesives does not contra-indicate subsequent surgical procedures in the event of a failure of UCF resolution with this simple therapeutic manoeuvre. The economic benefit of using adhesives to treat this complication appears to be significant, as it is the least expensive and most repeatable medical procedure without the risks of surgery. However, this assessment must be confirmed with an appropriate costing study on more cases.

## Abbreviations

ARR: Absolute risk reduction
FC: Fistula closure
HSR: Hypospadias surgical repair
NBCA: N-butyl-2-cyanoacrylate
NNT: Number needed to treat
OCA: 2-octyl cyanoacrylate
RRR: Relative risk reduction
UCF: Urethrocutaneous fistula.
